# The Western Journal

**Published:** 1843-12

**Authors:** 


### THE WESTERN JOURNAL.

Vol. VIII.—No. VI.

LOUISVILLE, DECEMBER 1, 1843.

### THE WESTERN JOURNAL.

This number completes our eighth volume, and we have determined to recommend to our publishers, to issue the first number of the next as the beginning of a new or second octade. This will afford a favorable opportunity for gentlemen wishing to subscribe, as they may commence with the series.

To the professional brethren who have made to us contributions of their experience, we tender thanks and acknowledgments. To those who have not, we take the liberty of saying that they *ought* to write. The profession has claims on all its members, which should be met. Physicians, as a body, compose a school of reciprocal instruction. They can look only to themselves and to each other for the means of improvement. Giving and receiving should then be mutual, although they may not be equal. Equality, indeed, is not either attainable or necessary in a system of interchangeable teaching. If we look to the East—to our elder brethren of the seaboard—and to the physicians and surgeons of Europe, for contributions to our magazines of knowledge, it is due to our own reputation, to common justice, and to the interests of humanity, that we should, as far as possible, maintain the balance of intellectual trade, by returning as much as may be in our power. The amount which might be given back, is, we are fully persuaded, far greater than is generally supposed; for the true character of the diseases of the various climates, and localities, and classes of people in the West and South, has not yet been made known. Now, every practitioner of medicine may, by recording and publishing, contribute something towards this good work.

Although we have spoken of the completion, by this number, of our *eighth* volume, our periodical has some claims to an earlier origin and much longer life. It is, in fact, a continuation of the *Western Journal*, begun in 1827, by one of the present Editors, and which, with some interruptions, was continued at Cincinnati, till 1839, when the subscribers were transferred to the publishers in Louisville. Thus it was the pioneer periodical of the West, or if not the very first, the only one that survived a single year. Perhaps it is not wholly unreasonable to claim for it a portion of respect on this account. If it has never been brilliant in style and speculation, nor a very *cornu copiæ* of experimental results, it has contained many communications that would have done honor to any periodical; with a still greater number which have contained useful observations on the diseases of the West; and has diffused such an amount of foreign intelligence, as to secure a *domestic* patronage, which has sustained it for sixteen years, during which nearly every eastern journal in existence at the time it was started has expired. We have underscored the word domestic, to indicate the locality of our subscribers. Almost without an exception they reside west of the mountains, between the Lakes and the Gulf of Mexico. Of the hundred and twenty professors, in the twenty schools, east of the mountains, but one is a subscriber; and out of those institutions we have but fourteen in that entire region. Is this the comity and consideration, which elder should extend to younger brethren, struggling under the disadvantages inseparable from a new community scattered through the wilderness? We wot not. Thus our journal does not divide the patronage of the East with its own; but the journals of that portion of the Union, compete for patronage with ours in the West. So far from complaining of this, it gratifies us much, as every exhibition of professional inquisitiveness always does. We would rejoice to see the time, when every medical man would subscribe for at least two journals. No one stands still in science. If not advancing, he is backsliding. But he cannot advance unless he combine the discoveries and inventions of his cotemporaries, with his own experience. As individuals, then, who cherish an abiding interest in the improvement of Western medicine, we would say to every medical gentleman—subscribe for at least one journal; make your selection, and if you should be disappointed in its character, drop it; but still subscribe—try another, and do not allow yourself to remain ignorant of the improvements which every day brings forth. It is the practice of many physicians, to wait till these improvements make their way into systematic works; but several objections lie against this custom. First, the delay; second, the condensed instead of the expanded statements of the original authors; third, the forced appropriation made by systematists and theorists, of new facts for the purpose of supporting their favorite hypotheses. The physician who relies on systems of surgery and physic for his information and intellectual discipline, acts unwisely—does his own mind injustice. To give it (in back-wood's phraseology), a fair chance, he should, as far as possible, learn the discoveries in the profession from their respective authors. In other words, the original extended account not only imparts the best information, but the study of it is the best exercise of the mind. He who relies upon compilations, may be a ready, but can never be a profound man. He adopts the results of inquiry without knowing the data from which they have been derived; has not, of course, travelled through the processes of logic by which they were obtained; and is unable to say, whether the conclusions he receives are true or false. Such should not be the information or mental discipline of him whose professional life, properly carried out, is one of unrelaxed mental exertion. But not intending to read a homily, we shall say no more on this topic.

We are happy to announce, that our publishers have made arrangements to print the next, and all subsequent numbers, on new type; and that each will be extended to ninety-six pages, making one hundred and forty-four pages more in the year, than heretofore, while the price will remain the same. This will enable us to give a more copious account of foreign discoveries and improvements than heretofore, and will we trust prove acceptable to all our subscribers.

